# Engagement and Stress Concentration Evaluation of a Novel Two-Part Compression Screw: A Preliminary Finite Element Analysis

**DOI:** 10.3390/bioengineering12050483

**Published:** 2025-05-01

**Authors:** Chia-Hao Hsu, Chih-Kuang Wang, Yan-Hsiung Wang, Sung-Yen Lin, Cheng-Chang Lu, Yin-Chih Fu, Hsuan-Ti Huang, Chung-Hwan Chen, Pei-Hsi Chou

**Affiliations:** 1 Graduate Institute of Clinical Medicine, College of Medicine, Kaohsiung Medical University, No. 100, Shih-Chuan 1st Road, Sanmin District, Kaohsiung 80708, Taiwan; ecowarrior.tw@yahoo.com.tw (C.-H.H.); hwan@kmu.edu.tw (C.-H.C.); 2 Department of Orthopedics, Kaohsiung Medical University Hospital, No. 100, Tzyou 1st Road, Sanmin District, Kaohsiung 80756, Taiwan; tony8501031@gmail.com (S.-Y.L.); cclu0880330@gmail.com (C.-C.L.); microfu@ms.kmuh.org.tw (Y.-C.F.); hthuang@kmu.edu.tw (H.-T.H.); 3 Department of Orthopedics, College of Medicine, Kaohsiung Medical University, No. 100, Shiquan 1st Road, Sanmin District, Kaohsiung 80708, Taiwan; 4 Orthopaedic Research Center, Kaohsiung Medical University, Kaohsiung 80708, Taiwan; ckwang@kmu.edu.tw (C.-K.W.); yhwang@kmu.edu.tw (Y.-H.W.); 5 Regenerative Medicine and Cell Therapy Research Center, Kaohsiung Medical University, Kaohsiung 80708, Taiwan; 6 Department of Medicinal and Applied Chemistry, College of Life Science, Kaohsiung Medical University, Kaohsiung 80708, Taiwan; 7 School of Post-Baccalaureate Medicine, College of Medicine, Kaohsiung Medical University, Kaohsiung 80708, Taiwan; 8 Department of Orthopedics, Kaohsiung Medical University Gangshan Hospital, Kaohsiung 820, Taiwan; 9 Department of Orthopedics, Kaohsiung Municipal Hsiao-Kang Hospital, Kaohsiung Medical University, Kaohsiung 812, Taiwan; 10 Department of Sports Medicine, College of Medicine, Kaohsiung Medical University, Kaohsiung 80708, Taiwan

**Keywords:** engagement assessment, finite element analysis, two-part compression screw, sleeve-nut screw, stress concentration, engagement percentage

## Abstract

**Background/Objectives:** This novel compression screw design offers potential benefits due to its two-part structure and can be used for various bone types, much like a conventional single-piece compression screw. However, full engagement may not always occur after final compression in clinical practice. This study aimed to verify the hypothesized optimal mechanical strength when the two parts are nearly fully combined and to determine a recommended engagement range based on stress distribution and concentration using finite element analysis. **Methods:** Ten models representing different combinations of the two screw parts (ranging from 10% to 100% of the engagement length, at 10% intervals) were simulated to determine the acceptable engagement percentage. Pull-out and bending load simulations were performed using finite element models. Extreme clinical loading conditions were simulated, including 1000 N pull-out forces and a 1 Nm bending moment at the screw head. **Results:** Finite element analysis revealed two stress concentration points. The pull-out load simulation showed that combinations with 100% engagement merged the two stress concentrations into one without force superposition, while combinations with less than 30% engagement should be avoided. In the bending load simulation, higher stress was observed for combinations with less than 90% engagement. A lower level of engagement increases the bending moment, which might be the major factor affecting the von Mises stress. **Conclusions:** Surgeons should be instructed on how to use the screw correctly and select the most appropriate screw size or length for the two parts to achieve an effective combination. Excessive bending or pull-out forces, or improper use with poor combinations, may cause the middle interval to strip or the screw to break or pull out. An engagement of more than 90% is recommended, while less than 30% is considered dangerous. This study provides biomechanical insights into this novel two-part screw design and its important clinical implications.

## 1. Introduction

The development of advanced fixation devices plays a crucial role in improving the outcomes of orthopedic surgery. Among these devices, compression screws have become essential for securing bone fragments and promoting bone healing by applying compressive forces to bone surfaces. Traditional single-piece compression screws are widely used in clinical practice [1,2,3,4], as are variable-pitch compression screws [5,6,7,8,9,10], both offering straightforward functionality. However, these single-piece screws have several potential limitations, including: (1) uneven force distribution during compression while rotating the screw, which may cause bone fragment movement [4]; (2) restricted compression length or low fragment compression, which cannot be applied to larger gaps; (3) a single opportunity for compression, which cannot be repeated; and (4) the inability to achieve optimal compression in certain clinical scenarios.

A novel two-part compression screw, also referred to as the sleeve-nut screw, has been introduced to address these limitations. This design incorporates two separate components—an inner screw and an outer sleeve—that work together to apply compression to the bone. The inner screw functions as a traditional compression screw, while the outer sleeve provides additional stability and allows for adjustments during the implantation process. This dual-component structure aims to provide better force distribution, more precise control over compression, and greater adaptability to various bone configurations compared to conventional single-piece screws. Recent studies on the sleeve-nut screw design, particularly its usage in odontoid fractures, show that it offers superior fragment compression and better resistance to screw stripping compared to classic compression screws [11,12]. These biomechanical studies suggest that two-part compression screws have the potential to improve the clinical outcomes of bone fixation, particularly in cases where traditional compression screws may fail to provide adequate engagement or compression. By using a sleeve-nut mechanism, the screw design offers more flexibility in adjusting compression after insertion, which can be particularly beneficial in complex fractures or in areas where precise bone alignment is critical. This ability to fine-tune compression during the procedure may help address inadequate compression and reduce complications, such as screw loosening, nonunion, pseudarthrosis, or implant failure, which are common issues with single-piece screw systems [4,6,10,13]. Furthermore, the modular nature of the design allows for greater customization of screw length and size, which is essential for accommodating variations in bone anatomy across different patients.

Despite these potential benefits, several challenges remain regarding the practical application of the two-part compression screw. Full engagement may not always occur after final compression in clinical practice. One key concern is achieving complete engagement between the two components during the implantation process, as incomplete engagement is hypothesized to lead to reduced mechanical stability. Moreover, the mechanical performance of the two-part engagement under various loading conditions, such as bending or shear forces, needs to be carefully evaluated to ensure its reliability and long-term durability. The use of finite element analysis (FEA) combined with additive manufacturing has proven valuable in orthopedic biomechanics, particularly for simulating implant performance and surgical planning. For example, 3D bio-models created via additive manufacturing have been used to evaluate von Mises stress distributions and osseointegration performance in intramedullary fixation systems for femoral fractures [14]. Additionally, rapid prototyping techniques have enabled the precise fabrication of patient-specific bone graft analogues, demonstrating the importance of computational modeling and simulation in enhancing biomechanical reliability and design accuracy [15]. Therefore, further finite element analysis or biomechanical studies are necessary to fully understand the advantages and limitations of the two-part compression screw in orthopedic surgery.

This study aims to provide biomechanical insights into the performance of the novel two-part compression screw by analyzing stress distribution patterns and evaluating the mechanical implications of different thread engagement percentages through finite element analysis. We hypothesize that a specific range of engagement percentages may offer improved mechanical performance and reduced stress concentration, compared to partial engagement configurations. By addressing these issues, this study seeks to contribute valuable data to the development and refinement of this innovative fixation device, ultimately improving patient outcomes in orthopedic procedures.

## 2. Materials and Methods

### 2.1. Two-Part Compression Screw Prototype Design and Implantation Procedures

**Figure 1:**
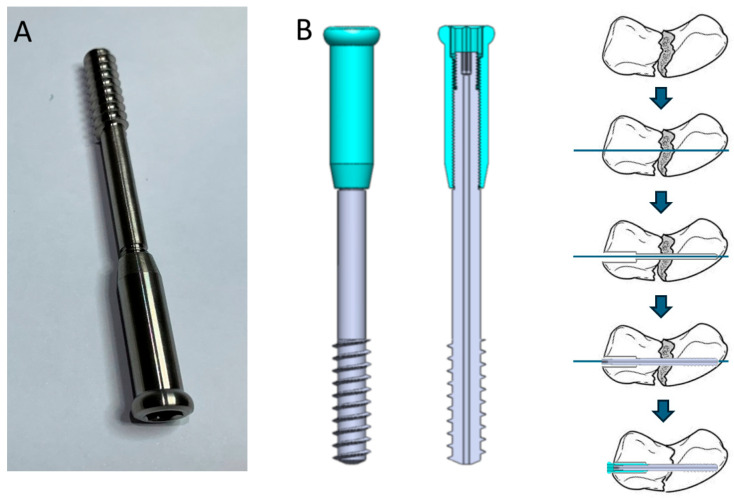
(**A**) Prototype of the novel two-part compression screw. (**B**) Schematic diagram of the two-part compression screw and implantation procedures.

The prototype of the novel two-part compression screw (Figure 1A) was created by the author. The original concept of this screw features a typical compression head with a sleeve nut, but it can also be combined with a headless sleeve nut for different types of clinical applications.

The schematic diagram shows that the two-part compression screw consists of two components (Figure 1B) connected by smaller-pitch full threads. In this prototype, the screw is designed as a cannulated screw, with each component featuring a StarDrive recess at the screw head for independent rotational control. The distal part has typical compression or lag screw-type threads, which are partially threaded. The implantation process begins with the insertion of a guide pin, followed by reaming for the main screw and proximal over-reaming for the sleeve nut. Next, the main screw is inserted, and the distal threads of the main screw are anchored into the far fracture fragment, functioning similarly to a lag screw in the metaphysis region. After connecting through the threads, the sleeve part, with a temporary washer-like tool, functions similarly to a push–pull reduction device when turned clockwise. Thus, the amount of reduction and compression can be adjusted freely by the surgeon using manual control. Fracture site compression can be achieved following the reduction procedure, and the tightness can be felt by the surgeon through mechanical feedback via the screwdriver.

Key factors for successful insertion include ensuring that the screw reaming and over-reaming of the sleeve nut area are sufficiently enlarged and accurately estimating the screw length. Fluoroscopic guidance can be beneficial for screw placement. However, the optimal sleeve nut length and the best method for combining the components still require further investigation. Thus, while the two-part compression screw shows potential benefits, further optimization and research are necessary to refine the technique.

Although a physical prototype of the two-part compression screw exists, investigating the appropriate combination percentage of the components is best achieved using finite element analysis (FEA). FEA enables the efficient simulation of various combination scenarios, offering valuable insights into stress distribution and mechanical behavior under different loading conditions. This approach is beneficial because it allows the evaluation of multiple configurations without the need for numerous physical prototypes. FEA is commonly used in the literature to assess screw performance and stress distribution in various orthopedic applications [16,17,18,19]. However, it is important to note that FEA simplifies model geometry and material properties, meaning it may not fully capture all the real-world complexities of the screw design.

### 2.2. Finite Element Analysis

**Figure 2:**
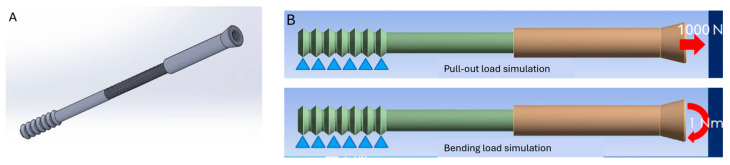
(**A**) Three-dimensional models of the screw were converted into finite element models. (**B**) Schematic illustration of the applied loading conditions, including a 1000-N axial pull-out force and a 1-Nm bending moment applied at the screw head to simulate extreme mechanical loading scenarios.

Three-dimensional models of the screw were converted into finite element models (Figure 2A). A mesh convergence test was performed to determine the optimal element size, and the model was considered converged when the change in peak von Mises stress between successive refinements was less than 5%. Based on this, the final mesh consisted of 18,520 20-node tetrahedral solid elements, which utilize higher-order interpolation to ensure accuracy near stress concentrations. The material properties of Ti6Al4V (elastic modulus: 113.8 GPa, Poisson’s ratio: 0.342, and yield strength: 790 MPa) were assigned to the screw elements, based on standardized data for orthopedic-grade titanium alloys [20]. To simulate clinically relevant loading scenarios, two extreme boundary conditions were applied at the screw head: a 1000-N axial pullout force and a 1-Nm bending moment (Figure 2B). These values were selected to represent upper-bound physiological loads encountered in osteoporotic bone or during accidental overloading. The distal tip of the screw was fully constrained in all degrees of freedom to replicate rigid fixation within cancellous bone. Ten models, representing different combinations of the two screw parts (ranging from 10% to 100% of the engagement length, at 10% intervals), were simulated to determine the acceptable engagement range for safe mechanical performance. The interface between the two screw parts was defined as a bonded contact, assuming complete thread interlocking without slippage or loosening, in order to isolate the structural response under idealized conditions. All simulations were performed using linear static structural analysis in ANSYS 7.0 (ANSYS Inc., Canonsburg, PA, USA). Material behavior was modeled as homogeneous, isotropic, and linearly elastic. Loading conditions were applied as constant, single-step loads rather than incrementally. Although this study did not include experimental validation, the modeling approach and parameter selection were informed by previously validated finite element frameworks used in orthopedic screw research [21,22,23], in which simulation outcomes have demonstrated strong agreement with mechanical test results. As such, the present results serve as a preliminary computational assessment of mechanical thresholds associated with varying thread engagement in two-part screw designs.

## 3. Results

### 3.1. Pull-Out Load Simulation with Different Two-Part Combination Percentages

**Figure 3:**
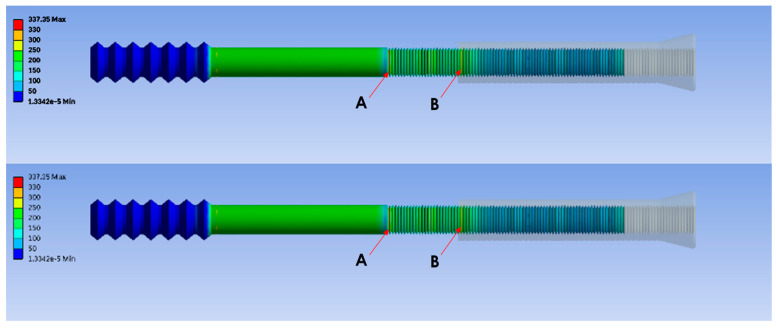
Finite element models showing two stress concentration points (Points A and B).

**Figure 4:**
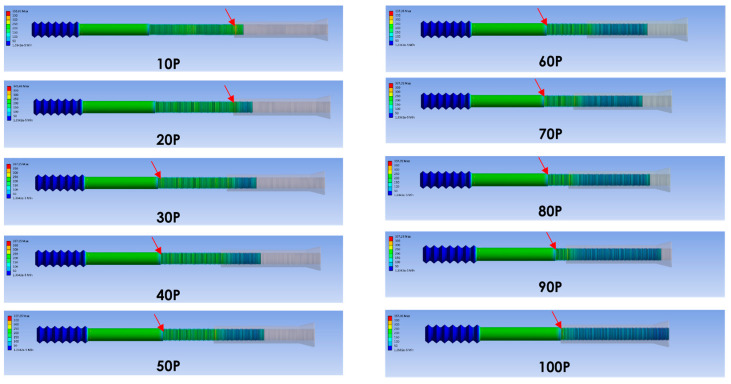
Pull-out Load Simulation in Finite Element Analysis. Ten models representing different combinations of the screw parts (ranging from 10% to 100% of the engagement length, at 10% intervals) were simulated. Red arrows indicate the point of highest stress. The major stress concentration point shifted from Point A to Point B when the combination was less than 30%.

The pull-out load simulation revealed two stress concentration points: one at the end of the middle thread (Point A, Figure 3) and the other on the middle thread at the end of the combination (Point B, Figure 3). Ten models representing different combinations of the screw parts (ranging from 10% to 100% of the engagement length, at 10% intervals) were simulated. The major stress was located at Point A. However, when the combination was less than 30%, the major stress concentration point shifted from Point A to Point B (Figure 4). At a 100% combination, Points A and B merged into one.

**Figure 5:**
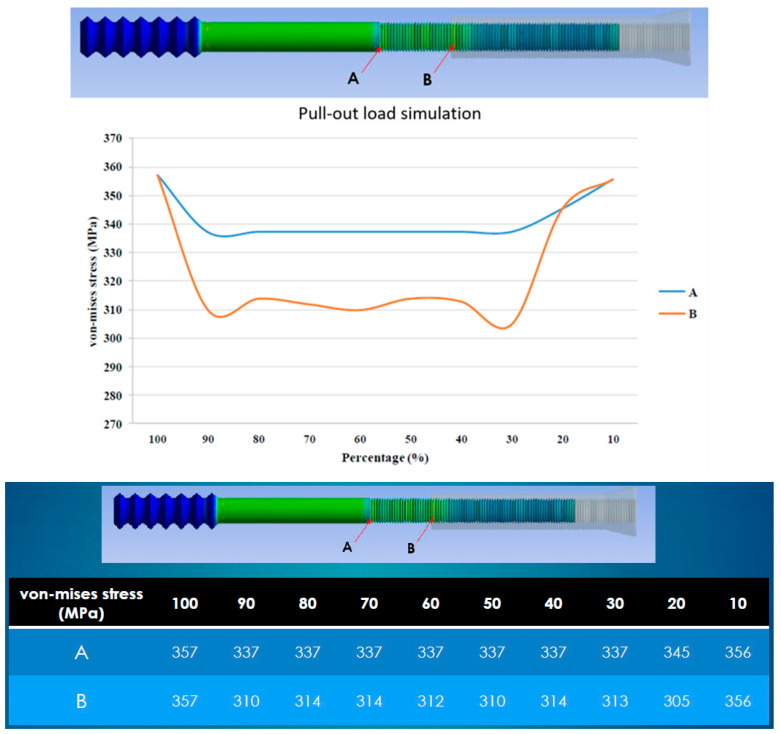
Von Mises Stress at Points A and B for Different Combinations in the Pull-out Load Simulation. The safer range of combination is suggested as at least 30%.

According to the results, we suggest that a combination of at least 30% or more is the safer range (Figure 5). However, because we used 10% intervals, this range may not be sufficiently precise. In other words, combinations of less than 30% should be avoided. A higher von Mises stress (357 MPa) was observed when the length of engagement was 100%. This was likely to be due to the merging of the two points into one, but without any obvious force superposition or stress doubling (Figure 5).

### 3.2. Bending Load Simulation at Different Two-Part Combination Percentages

**Figure 6:**
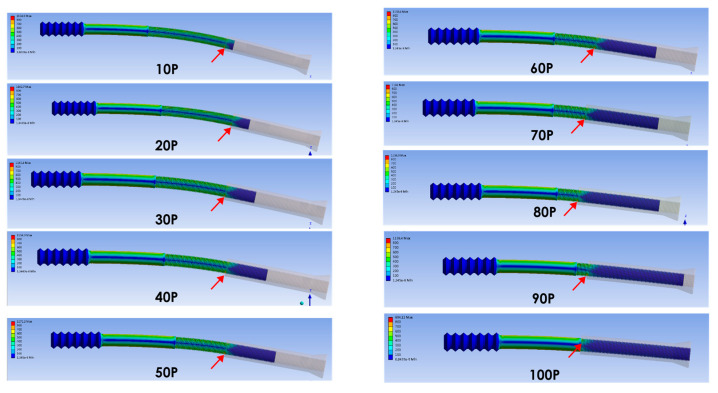
Bending load simulation in finite element analysis. Ten models representing different combinations of the two screw parts (ranging from 10% to 100% of the engagement length, at 10% intervals) were simulated. Red arrows indicate a clear transition between high and low stress, which corresponds to Point B.

In the bending load simulation of the finite element analysis, ten models representing different combinations of the screw parts (ranging from 10% to 100% of the engagement length, at 10% intervals) were simulated. Red arrows indicate a clear transition between high and low stress, which corresponds with Point B (Figure 6).

**Figure 7:**
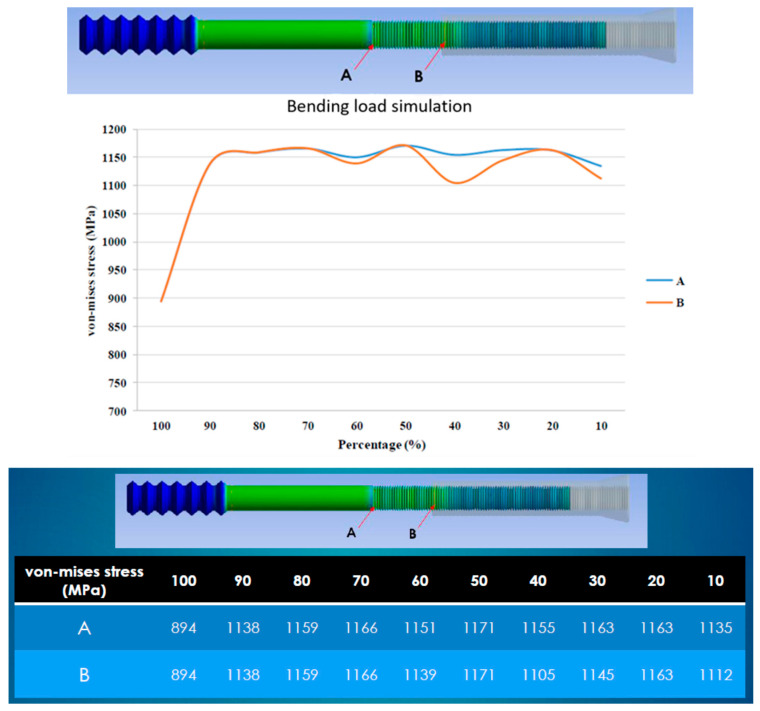
Stress at points A and B for different combinations in the bending load simulation. The length of engagement did not influence the stress distribution for combinations between 10% and 90%. The optimal combination is suggested to be greater than 90% to minimize the potential bending force. Higher von Mises stress, exceeding 1000 MPa, occurred at both Point A and Point B when the engagement length was less than 90%.

Higher stress was observed for combinations less than 90%. The moment arm was likely to be the major contributing factor. Therefore, we suggest that a combination greater than 90% results in a lower bending moment. However, the von Mises stress obtained was similar for combinations between 10% and 90%, which did not correlate with the engagement length. In summary, the length of engagement did not significantly affect the stress distribution, which remained relatively consistent under bending forces (Figure 7). The bending load simulation revealed that higher von Mises stress, exceeding 1000 MPa, occurred at both Point A and Point B when the engagement length was less than 90%.

Minor variations in von Mises stress observed at locations A and B during the bending load simulation (Figure 7) may be attributed to two key factors. First, changes in the threaded interface condition across models lead to structural redistribution of the bending moment, thereby altering local stress concentration patterns. Second, despite performing a mesh convergence analysis, slight numerical fluctuations may persist due to the complex contact mechanics at the thread interface, which are typical in finite element simulations. These variations remain within acceptable limits and do not affect the overall trend or interpretation of the mechanical performance.

It should be noted that the bending load applied in the simulation was intentionally set at a higher level to evaluate the screw’s mechanical limit under extreme conditions. As a result, von Mises stress values exceeded the yield strength of the material, indicating potential plastic deformation under such loading. These findings are not indicative of routine clinical loads but rather serve to evaluate design safety under worst-case scenarios.

## 4. Discussion

This study focused on how the mechanical performance of a two-part screw is influenced by varying degrees of thread engagement. While general concepts of threaded engagement and bolted joint behavior have been described in the field of mechanical engineering—including engagement length optimization [24,25] and the influence of shear stiffness and loading [26]—there remains a lack of clinically oriented guidance for surgical applications involving novel two-part screw designs. Our finite element approach aimed to simulate real-world deviations in which full engagement may not be achievable due to anatomical or procedural constraints. While analytical beam theory provides basic insights into deflection under ideal conditions, it is insufficient to address the complexity of this novel two-part screw system. Rather than proposing a new theoretical framework, our goal was to identify the safe functional range of partial engagement within a surgical context. This perspective bridges the gap between mechanical design knowledge and the practical needs of orthopedic surgeons working with two-part compression screws.

Moreover, it is important to note that this study did not explicitly simulate bolt preload or tightening torque—factors known to significantly influence screw performance and the risk of loosening. Bolt preload generates axial clamping force that maintains contact and friction between engaged threads, thereby preventing micromotion and subsequent loosening [27]. The omission of preload modeling is acknowledged as a limitation of this work, and future studies should incorporate this factor to better approximate clinical conditions. We believe that including torque-preload relationships and frictional thread behavior will offer a more comprehensive biomechanical understanding and more closely approximate clinical reality.

It is important to clarify that the loading conditions used in the simulation were selected to represent extreme clinical scenarios rather than typical physiological loading. These include situations such as early weight-bearing, malposition during insertion, or traumatic postoperative events. Such loads were intended to test the mechanical limits of different engagement combinations and identify failure thresholds under worst-case conditions. This methodology is commonly used in preliminary implant assessment to ensure robustness under high-stress environments, even though it may not capture the full range of daily in vivo forces.

To the best of our knowledge, this is the first study to present and delineate the combination parameters of the novel two-part compression screw. In the literature, the design most similar to our two-part compression screw is the sleeve nut screw, a cannulated spongiosa screw with a shaft and sleeve nut (Signus Medizintechnik GmbH, Alzenau, Germany) [11,12]. However, there are currently very few studies discussing this sleeve nut screw design, and its usage is limited to odontoid fractures. One study from 2020 indicated that lag screw osteosynthesis in odontoid fractures shows a high rate of pseudarthrosis. Biomechanical factors may play a role in this, such as insufficient fragment compression or unnoticed screw stripping. The biomechanical comparison revealed that the double-threaded screw is robust against screw stripping but provides only low fragment compression. In contrast, classic compression screws offer better compression but are more sensitive to screw stripping. The sleeve-nut screw outperforms in compression and is as robust as the double-threaded screw against screw stripping [11]. Another study from 2024 demonstrated that screws with sleeve nuts provide greater fragment compression and are more resistant to screw stripping compared to classic compression screws [12].

Although a physical prototype of the two-part compression screw has already been developed, investigating the most effective combination percentage of the two parts remains crucial for achieving the best mechanical performance in clinical practice. In practical terms, the most effective way to explore this relationship is through FEA. FEA is widely utilized in recent orthopedic research to evaluate screw performance, including stress distribution, pull-out strength, and fracture fixation stability under various clinical scenarios. Studies have modeled different screw geometries, thread designs, and loading conditions to better understand how implant parameters influence mechanical outcomes [16,17,18,19]. These applications demonstrate the value of FEA not only as a validation tool but also as a design optimization method for novel orthopedic implants. FEA allows for detailed simulation and evaluation of the screw’s mechanical behavior under various loading conditions, which can be difficult to replicate experimentally due to the complexity of bone–screw interactions. Using FEA, it is possible to model different combination percentages of the two components—inner screw and outer sleeve—and assess their impact on stress distribution, force transmission, and overall mechanical stability without the need for multiple physical prototypes. This computational approach offers several advantages, including the ability to explore a wide range of scenarios in a controlled, cost-effective manner. Additionally, FEA enables the visualization of stress concentrations and potential failure points, providing insights into areas that would be challenging to observe through traditional physical testing. While classical beam theory can approximate deflection under simplified conditions, it is insufficient to capture the complex geometry, contact behavior, and localized stress distribution of the two-part screw. In contrast, FEA allows for a more accurate simulation of partial engagement, internal thread contact, and stress gradients that cannot be resolved analytically. However, it is important to note that finite element models involve simplifying certain aspects of the system. Specifically, the models often use idealized geometries and assumptions regarding material properties and boundary conditions, meaning that they may not fully capture the intricacies of the real-world design. While this simplification is necessary to make the analysis computationally feasible, it may not perfectly replicate every aspect of the physical screw, including minute geometric variations or material inconsistencies that could influence the performance under real-world conditions. Nonetheless, FEA remains the most suitable tool for understanding the mechanical behavior of the two-part compression screw and refining its design for practical application.

This study used FEA to evaluate the performance of a novel two-part compression screw design under extreme clinical loading conditions. The results revealed two key stress concentration points during the pull-out and bending load simulations, providing valuable insights into the optimal engagement configuration for the screw parts. In the pull-out load simulation, combinations with 100% engagement resulted in the merging of the two stress concentration points into one, without noticeable force superposition. This suggests that fully engaged screws may offer a more uniform stress distribution and greater stability during pull-out forces. Conversely, combinations with less than 30% engagement exhibited a shift in the major stress concentration point from one location to another, indicating an increased risk of screw failure. These findings suggest that a higher engagement percentage is crucial for maintaining the screw’s mechanical integrity, and combinations with less than 30% engagement should be avoided to prevent premature failure or instability. The bending load simulation revealed an interesting trend: lower engagement levels (below 90%) led to higher stress levels. This was attributed to an increased bending moment at the screw head, which in turn amplified the von Mises stress. These results indicate that insufficient engagement compromises the screw’s ability to resist bending forces, potentially leading to bending failure or deformation under clinical loading conditions.

This study’s findings highlight the importance of selecting an appropriate combination percentage to balance both pull-out and bending resistance. Specifically, the data suggests that a combination percentage above 90% ensures greater mechanical stability and is less prone to failure due to bending or pull-out forces. These insights are crucial for improving the clinical performance of two-part compression screws, particularly in applications that require high resistance to mechanical forces, such as fracture fixation. These findings not only clarify the biomechanical behavior of partial-thread engagement but also offer a theoretical basis for future development of modular orthopedic implants. Although this two-part compression screw has not yet been widely applied in current bioengineering or clinical practice, the present simulation-based analysis aims to establish a biomechanical foundation for future experimental and clinical implementation.

Currently, there are no similar studies in the literature that specifically focus on the combination parameters of two-part compression screws in the context of both pull-out and bending forces under extreme loading conditions. While there are numerous studies examining single-screw designs or general screw mechanics, this study provides a unique contribution by exploring the effects of different engagement combinations and offering biomechanical evidence for optimizing screw design. The absence of comparable studies further emphasizes the novelty of our approach and the potential clinical significance of our findings in improving screw performance in orthopedic applications.

Compared to the conventional one-piece compression screw, our new screw design incorporates an additional middle thread interval between the two parts. This interval provides mild, limited flexibility between the components, potentially reducing stiffness in a manner similar to semi-rigid screws, although it is not designed for locking purposes, as in far cortical locking screws [28,29,30,31,32,33,34,35,36,37,38,39,40] or dynamic locking screws [41,42,43,44,45,46], which may allow micromotion at the fracture site and promote natural bone healing [47]. While reducing stiffness, we believe our construct remains sufficiently strong and not overly “soft”, as the middle thread provides an additional thread-to-thread and metal-to-metal locking combination. However, whether the potentially reduced stiffness leads to a decrease in nonunion or pseudarthrosis remains to be verified in clinical studies. In addition to stiffness-related issues, one-piece compression screws often fail to achieve full compression due to their limited compression length, resulting in a high-strain environment at the fracture site and excessive stress on the implant. The residual fracture gap may remain after the one-piece compression screw is fully inserted, potentially leading to nonunion or construct failure. Another potential advantage of this novel two-part compression screw is that the tightness of the compression and the stability of the threads fixed inside the fragment can be felt and confirmed by the surgeon. Additionally, the fixation depth can be freely controlled using two independent screwdrivers: one for the main screw and the other for the sleeve nut.

Our study has several limitations. First, the study relied on FEA to simulate the behavior of the screw under extreme loading conditions. While FEA is a powerful tool for predicting mechanical performance, it is based on certain assumptions and simplifications that may not fully capture the complexity of real-world clinical conditions. For example, the modeling did not account for biological factors such as bone quality, healing progress, or the influence of soft tissue, all of which may affect screw performance in vivo. Second, the simulated extreme clinical loading conditions may not represent the full spectrum of typical clinical scenarios, where forces are often lower, more varied, and time-dependent. While these high-load conditions are useful for identifying worst-case failure points, future studies should incorporate more realistic, dynamic, and patient-specific loading conditions to better approximate in vivo performance. Third, clinical validation is required to assess its real-world effectiveness and safety in combination. The potential impact of reduced stiffness and improved reduction/compression on outcomes such as reduced nonunion and pseudarthrosis remains to be proven in clinical trials. Fourth, the study modeled only ten variations of the screw combination, which may not fully represent the variety of real-world scenarios where the screw’s design could vary. Additionally, the study’s focus on a single screw design may not account for the geometric diversity found in different types of fractures or anatomical variations. Further research with a broader range of screw models and fracture types is needed to generalize the results. Fifth, the finite element model did not incorporate bolt preload or thread friction behavior, both of which are known to significantly influence the mechanical performance of screw joints. The omission of preload modeling means that the clamping force and its effect on micromotion and screw loosening were not assessed. Future simulations that include torque–preload interactions and thread interface mechanics would offer a more comprehensive biomechanical analysis and better reflect clinical conditions. Sixth, although a mesh convergence test was performed based on changes in peak von Mises stress, a comprehensive mesh sensitivity analysis was not conducted. Additional parameters—such as displacement and contact pressure—were not evaluated across varying mesh densities. While the selected mesh was adequate for stress-based analysis, this limitation may affect the accuracy of other mechanical predictions. Seventh, while the current study provides a computational evaluation of screw performance, direct experimental validation of the finite element model was not performed. Although the simulation framework was informed by previously validated FEA approaches, confirmation through mechanical testing remains essential. Future studies will involve bench-top experiments using a physical prototype of the two-part screw to verify the accuracy and clinical relevance of the numerical findings. Eighth, no direct comparison was made with analytical equations from classical mechanics or experimental data, due to the novel and geometrically complex structure of the two-part compression screw. While classical beam theory provides general insights, it cannot capture the localized stress patterns and interfacial mechanics inherent to this design. Additionally, no physical testing of this prototype has been performed to date. Future studies should incorporate analytical approximations under simplified conditions and conduct mechanical experiments to validate and complement the numerical findings.

## 5. Conclusions

This study is the first to investigate the mechanical behavior of different engagement configurations in a novel two-part compression screw using finite element analysis. The simulation results demonstrated that stress concentrations during pull-out and bending load conditions are significantly influenced by the degree of engagement between the two screw parts. Combinations with less than 30% engagement exhibited high stress and poor mechanical stability, while combinations above 90% showed favorable stress distribution and reduced risk of failure. Based on these findings, a minimum engagement threshold of 90% is recommended to ensure mechanical safety and performance.

Clinically, surgeons should be guided in selecting the proper screw size and ensuring sufficient engagement during implantation to minimize complications, such as screw breakage or loosening. Future research should include mechanical testing to validate these numerical findings, as well as clinical studies to assess the long-term outcomes of using this two-part screw system in different anatomical and loading conditions.

## Data Availability

The data presented in this study are available on request from the first author.
